# *Thitarodes shambalaensis* sp. nov. (Lepidoptera, Hepialidae): a new host of the caterpillar fungus *Ophiocordyceps sinensis* supported by genome-wide SNP data

**DOI:** 10.3897/zookeys.885.34638

**Published:** 2019-11-04

**Authors:** Zhengyang Wang, Hailing Zhuang, Min Wang, Naomi E. Pierce

**Affiliations:** 1 Department of Organismic and Evolutionary Biology, Harvard University, 26 Oxford Street, Cambridge, MA 02138, USA Harvard University Cambridge United States of America; 2 Department of Entomology, College of Agriculture, South China Agricultural University, 483 Wushan Road, Guangzhou, 510642, China South China Agricultural University Guangzhou China

**Keywords:** RAD-Seq, phylogeny, new species, caterpillar fungus, Kangding, Mt. Gongga, Sichuan, China

## Abstract

A new species of ghost moth, *Thitarodes shambalaensis***sp. nov.**, is described from Yanzigou glacier, Mt. Gongga, Sichuan, China. The species is a host of the economically important caterpillar fungus *Ophiocordyceps sinensis*. Establishment of this new species is supported by morphology and genetic differentiation measured in a CO1 phylogeny and in genome-wide SNP coverage. A summary tree from 538 sequences of different genetic markers from *Thitarodes* (including sequences extracted from caterpillar fungus sclerotium samples) support the genus *Thitarodes* as a monophyletic group, and indicate that *Thitarodes* is the host genus for *O. sinensis*. Sampling efforts so far have centered on half of the known phylogenetic diversity of *Thitarodes*, with some species-level clusters (separated by < 2.5% genetic distance) containing 17 described species. Fifteen clusters are known from either a single “orphan taxon” or a single sequence from a caterpillar fungus sclerotium sample. We provide suggestions for building a more robust phylogeny of the genus *Thitarodes* and highlight some of the conservation threats that species from this genus face due to unprecedented habitat exploitation.

## Introduction

The genus *Thitarodes* Viette, 1968 of the ghost moth family Hepialidae was first established by Viette,1968 to accommodate *Hepialus armoricanus* Oberthür, 1909 found in China and three other new species found in Nepal (*T. danieli* Viette, 1968, *T. eberti* Viette, 1968, and *T. dierli* Viette, 1968). Chu (1965) was the first to show that *T. armoricanus* and other species of this genus are the hosts of caterpillar fungus: the larvae of which are often parasitized by the entomophagous fungus *Ophiocordyceps sinensis* (Berk.) G.H. Sung et al. (family Ophiocordycipitaceae) (see Sung et al. 2007 and review by Shrestha et al. 2014). The infection of *Thitarodes* larvae by *O. sinensis* produces a sclerotium consisting of hyphal threads enclosed within the larval exoskeleton. This sclerotium, known as caterpillar fungus, is coveted in eastern Asia, particularly China, as a traditional herbal medicine (Winkler 2008, 2010) and is in decline due to both overharvesting and climate change (Hopping et al. 2018).

After Chu’s (1965) discovery of *T. armoricanus* as the host for caterpillar fungus, many new ghost moth species were subsequently described in the 1980s by workers at the Chinese Academy of Sciences under the genus *Hepialus* Fabricius, 1775 (see review by Wang and Yao 2011). These taxa were transferred to *Thitarodes* in the global revision of infraorder Exoporia by Nielsen et al. (2000). More recently, eight new species have been described by Chu et al. (2004) under *Hepialus*. A total of ten new species has been described by different authors under *Thitarodes* (Ueda 2000; Zhang et al. 2007; Maczey et al. 2010; Zou et al. 2011; Jiang et al. 2016).

The inventory by Nielsen et al. (2000) listed 51 species under *Thitarodes* worldwide, while Chu et al. (2004) listed 54 species of this genus in China alone. The review of Wang and Yao (2011) estimated that only 37 of the described *Thitarodes* species are potential hosts of the entomophagous fungus *O. sinensis*. The inconsistencies in the number of species in *Thitarodes* underscores several difficulties in the taxonomy of this group:

1. *Thitarodes* adults are difficult to collect. Both Ueda (2000) and Zou et al. (2011) have mentioned that adults of this genus do not come readily to light and have a short active period around sunset. Ueda (2000) estimated that adults of some species are only active for about a week annually. Tao et al. (2015) conducted laboratory rearing studies where the time to complete the developmental cycle from egg to adult was 494 and 780 days for *T. jianchuanensis* (Yang, 1994) and *T. armoricanus*, respectively. Larvae of the genus are subterranean root-borers. The short window for collecting adults is further hindered logistically by the inaccessibility of the species habitat at high elevation (> 3000 m) valleys of the Himalaya and the Tibetan plateau.

2. Since most *Thitarodes* species have few distinctive wing scale patterns, new species descriptions are based primarily on male genitalia, sometimes coupled with a description of wing venation. Moths of the infraorder Exoporia, which contain the genus *Thitarodes*, are distinguished by the unique configuration of female genitalia: in female exoporian Lepidoptera, sperm is transferred to the egg via an external seminal gutter. This important feature has been largely ignored in descriptions of the genus *Thitarodes*. After re-examining the holotype of *T. armoricanus*, Ueda (2000) concluded that the male holotype designated by Oberthür (1909) was in fact a female; thus, the identity of *T. armoricanus* is problematic without a male holotype. Although some authors describe *Thitarodes* larvae, pupae and adult leg morphology in detail (Ueda 2000; Chu et al. 2004), these traits have not been included in the classification key proposed by Chu et al. (2004).

3. Several *Thitarodes* species described before 2000s were only known by drawings of wing venation and genitalia structure. Access to the holotypes of these species is limited. Many species are only known from a few samples collected in restricted localities (such as *T. zhongzhiensis* (Liang, 1995) in “the middle of Renzhi snow mountain”, *T. anomopterus* (Yang, 1994) on the “north-west slope of Mt. Laojun, Yunnan” , *T. jialangensis* (Yang, 1994) in “Jialang county west of Meili mountain”), and have not been further studied (Yang 1994; Liang 1995). This has made comparative analysis across different species of the genus *Thitarodes* difficult. An effort at revising the genus (Zou et al. 2010) divided *Thitarodes* into four genera, but for this paper, we follow the taxonomy of Nielsen et al. (2000).

In recent decades, molecular evidence has helped resolve difficult problems in taxonomy, including elucidating the backbone of non-ditrysian Lepidoptera including Hepialidae (Regier et al. 2015). Although a few sequences of described species of *Thitarodes* have been contributed to GenBank by different authors (see Suppl. material 1: Table S1 and Suppl. material 3: Table S3), not all sequences have been published, and thus the validity of species identifications cannot be verified. A molecular phylogeny for the genus *Thitarodes* has not yet been published. This is likely due to the difficulty of obtaining genetic material from previously described species, either from museum loans or from the field. Creatively, most of the molecular approaches to resolving the taxonomy of the genus *Thitarodes* has taken the unorthodox route of sequencing genetic material of *Thitarodes* from widely-available samples of caterpillar fungus (Zhang et al. 2014; Quan et al. 2014a, b). This method bypasses the difficult task of sampling adult *Thitarodes* moths at high altitude. Comparing genetic material of *Thitarodes* moths and *Ophiocordyceps* fungi from the same caterpillar fungus sclerotium also provides insight into host-parasite coevolution across a wide geographical range. The drawback of this approach is that the sequences of *Thitarodes* obtained from caterpillar fungus typically have no voucher specimens of either adult moth or mummified caterpillar; unless matched with an identical (or extremely similar) sequence from a known species. It is thus difficult to assign the sequences from caterpillar fungus to a particular moth species.

While molecular work using samples of caterpillar fungus sclerotia has only yielded at most three DNA fragments with which to evaluate each sample, the development of next-generation sequencing techniques has offered researchers new genotyping-by-sequencing methods to obtain large numbers of loci from non-model organisms for phylogenetic studies (Andrews et al. 2016). Restriction site Associated DNA sequencing (RAD‐Seq) (Davey and Blaxter 2010) can generate genetic markers across many individuals at a reasonable cost. This is achieved by sequencing genomes of reduced complexity using restriction enzyme digestion. As the technique was first designed to identify SNPs at the within-species level, using RAD‐Seq to sequence a population of individuals from multiple species might result in a reduced number of intra-species loci (Cariou et al. 2013; Eaton et al. 2017). This is because if the level of genetic divergence among individuals exceeds what is typical at the population level, this will reduce the number of conserved restriction sites the enzymes can target. We would expect highly diverged species pairs with long branch lengths to share few loci from RAD‐Seq. This can often be used to identify samples from distinct lineages (for a visualization tool see de Medeiros and Farrell 2018).

In this paper we describe *T. shambalaensis*, a new species in the genus *Thitarodes* from the Yanzigou glacial valley, Mt. Gongga, Sichuan, China, including analysis of wing venation, male genitalia, labial palps, phenology and habitat. We discuss some of the conservation threats this species is facing due to recent habitat exploitation related to local experimentation on caterpillar fungus-farming. We evaluate the validity of *T. shambalaensis* as a new species by providing data from morphology, CO1 sequences (i.e., constructing a phylogenetic tree with all known CO1 sequences of *Thitarodes*) and genome-wide SNP sequences (i.e., comparing intra and inter-species SNP sequence coverage), and we infer a “summary tree” from all known sequences of different genetic segments of *Thitarodes*, both from sequence deposits with known species names and from sequences of caterpillar fungus sclerotia (Suppl. material 3: Table S3). We show that sequences of sclerotia can be matched with sequences from adult *Thitarodes* samples with identifiable names, and that these sequences together constitute a well-established monophyletic group (the genus *Thitarodes*). We briefly evaluate the taxonomic decision of Zou et al. (2010) to split *Thitarodes* into four different genera, highlight gaps in our knowledge from uneven sampling across the different clades revealed by our phylogeny and provide suggestions for building a more robust phylogeny of the genus *Thitarodes*.

## Materials and methods

### Collection, preservation, and description

All adult samples described were collected between June and July 2016, at Yangliuping (29°41'2.54"N, 101°53'32.24"E), inside Yanzigou glacier, Mt. Gongga, Sichuan (Fig. 2, Fig. 8). Since *Thitarodes* species do not congregate at light traps and mating flights have not been observed for this particular species, collection was undertaken by thoroughly searching through habitat vegetation with flashlights. Adults can be found hanging at the edge of low vegetation, especially near where pupae molts can be seen. Collection for adults was also attempted at other glacial valleys along eastern Mt. Gongga from June to July between 2016 and 2018 but were unsuccessful. Pupae and larvae were collected at multiple glacial valleys along eastern Mt. Gongga (Fig. 8, see Suppl. material 2: Table S2 for names of seven valleys and their coordinates) as well as a habitat east of the Kangding-Moxi fault (Yajiageng, 29°53'53.12"N, 102° 0'37.87"E) from May to June, 2016 to 2018, with assistance from the local communities (see Acknowledgments). In each valley, at habitats where caterpillar fungus is harvested by local people, multiple 50 × 50 × 30 cm grassland plots were searched to discover ground-boring *Thitarodes* larvae and pupae. Grass coverage were placed back after sampling. Samples were preserved in 90% ethanol. Photographs of specimens were taken with a Nikon Coolpix 4500 digital camera. Dissections were performed after softening adult genitalia in heated 10% NaOH solution for 10–30 mins and transferring the genitalia on dissection slides with glycerol. Wing venation slides were obtained by softening the wings in 30% dish detergent for 30–60 mins, and gently brushing the scales off the wings. Slides were examined and photographed under a Carl Zeiss Stemi 2000-CS stereoscope system and Carl Zeiss SteREO Discovery V12 stereoscopic microscope system. Although previous authors have used the Comstock-Needham venation nomenclature to describe new species (e.g., Maczey et al. 2010; Zou et al. 2011), Wootton’s venation nomenclature (1979) was used in our description to reflect differences in wing homology.

### DNA extraction, sequencing, and analysis

Genomic DNA of 134 samples of *Thitarodes* were extracted from leg (adult) or thoracic (larvae, pupae) tissue with Qiagen DNeasy Blood and Tissue Kits (Qiagen Inc.). The COI region of 48 samples was amplified and sequenced with LCO1490 (Folmer et al. 1994) and Nancy (Bogdanowicz et al. 1993) primers, following the protocol for Lepidoptera COI sequencing outlined in Boyle et al. (2014) (with the exception that the annealing temperature was set at 55 °C, repeating for 35 cycles). Samples were sequenced at Thermo Fisher Scientific Inc, Shanghai. These sequences were aligned in Geneious Prime (using the MAFFT algorithm) together with nine other COI sequences of described *Thitarodes* species in China and available on GenBank (five of which are segments of mitogenome sequences). Outgroups were selected according to Grehan (2011, 2012). We used either COI sequences or the COI segment of the mitogenome sequence of the East Asian genus *Napialus* Chu & Wang, 1985, the Neotropical genus *Phassus* Walker, 1856, and the Australian genus *Oxycanus* Walker, 1856 (Hepialidae) (see Suppl. material 1: Table S1 for all sequences used). A Maximum Likelihood tree was inferred using the IQ-Tree algorithm (Nguyen et al. 2015) with a free rate variation model (Kalyaanamoorthy et al. 2017) and ultrafast bootstrap (UFBoot, Hoang et al. 2018) integrated 1000 times and SH-aLRT branch test (Guindon et al. 2010). All 134 extracted DNA samples were digested with EcoRI enzyme and library preparation followed the wet lab protocol of Etter et al. (2012). Samples were sent for 150bp pair-end sequencing on an Illumina HiSeq4000 platform located at 1gene Inc, Hangzhou. Illumina reads were demultiplexed and processed by STACKS (Rochette and Catchen 2017). For each sample, a locus was identified with a minimum depth coverage of three reads, and a 2bp distance was allowed between each locus. Loci having a distance less than 1bp across individuals were merged into consensus loci. The resulting matrix of all SNPs from all samples was converted into a presence/absence matrix, where missing reads (indicative of divergent enzymatic cut sites) were noted by 0 and otherwise 1, as in de Medeiros and Farrell 2018.The resulting matrix rows were arranged by hierarchical clustering (Ward 1963) to facilitate visualization.

### Summary tree

A total of 541 available sequences of *Thitarodes* was used to build a summary tree. These sequences came from three sources: CO1 and mitogenome sequences of known *Thitarodes* species used in generating the Maximum Likelihood CO1 tree in this study (Suppl. material 1: Table S1), 26 Cytb sequences from known *Thitarodes* species as described in Zou et al. (2017), 226 published sequences of different segments extracted from caterpillar fungus sclerotia (Zhang et al. 2014; Quan et al. (2014a, b). The latter sequences were not associated with clear species names. CO1, Cytb and wg sequences from a single sample in Zhang et al. (2014) were concatenated, and CO1, Cytb and CO2 sequences from a single sample in Quan et al. (2014a, b) were concatenated. All sequences, after concatenation, were aligned in Geneious Prime (MAFFT algorithm). For mitogenome sequences, only the CO1, CO2 and Cytb segments were used in alignment. The Maximum Likelihood tree was inferred the using the same method CO1 sequence tree was inferred (see previous section). In the resulting phylogeny, each tip within 0.025 distance from each other were clustered and labelled as a single cluster to assess the degree of uneven sampling across the phylogeny.

## Taxonomy

### Thitarodes shambalaensis sp. nov.

Taxon classificationAnimaliaLepidopteraHepialidae

FF2E126C-37A0-56E8-8512-31A1FEA7C6DE

http://zoobank.org/DADDC567-C706-4857-800A-B736C4719774

**Figure 1.**
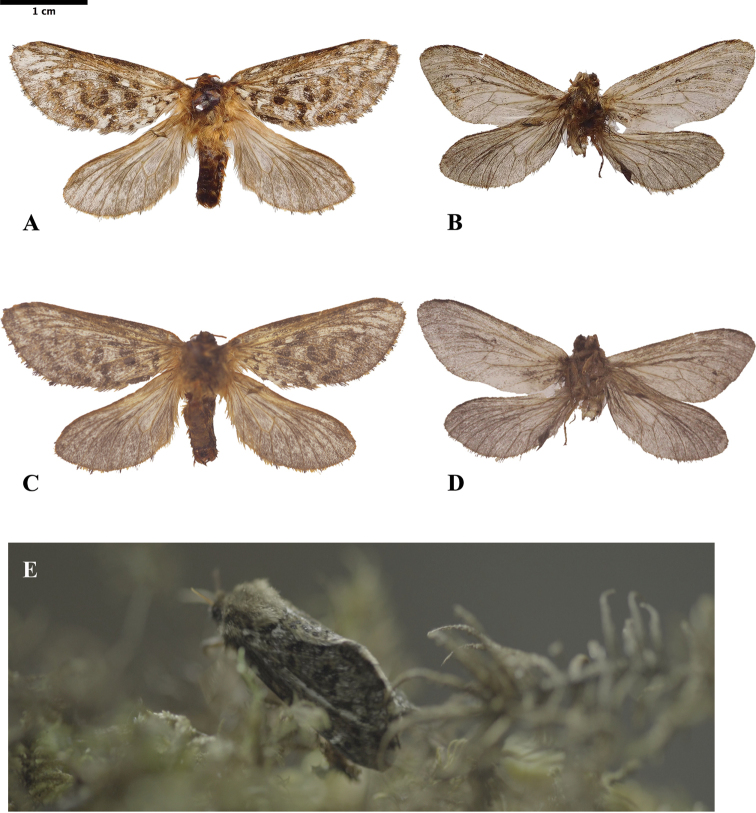
*Thitarodes shambalaensis* sp. nov. **A** holotype, male, dorsal view **B** paratype, female, dorsal view **C** holotype, male, ventral view **D** paratype, female, dorsal view **E** resting position in habitat (photograph credit Hua Zhang). Scale bar: 1 cm.

**Figure 2.**
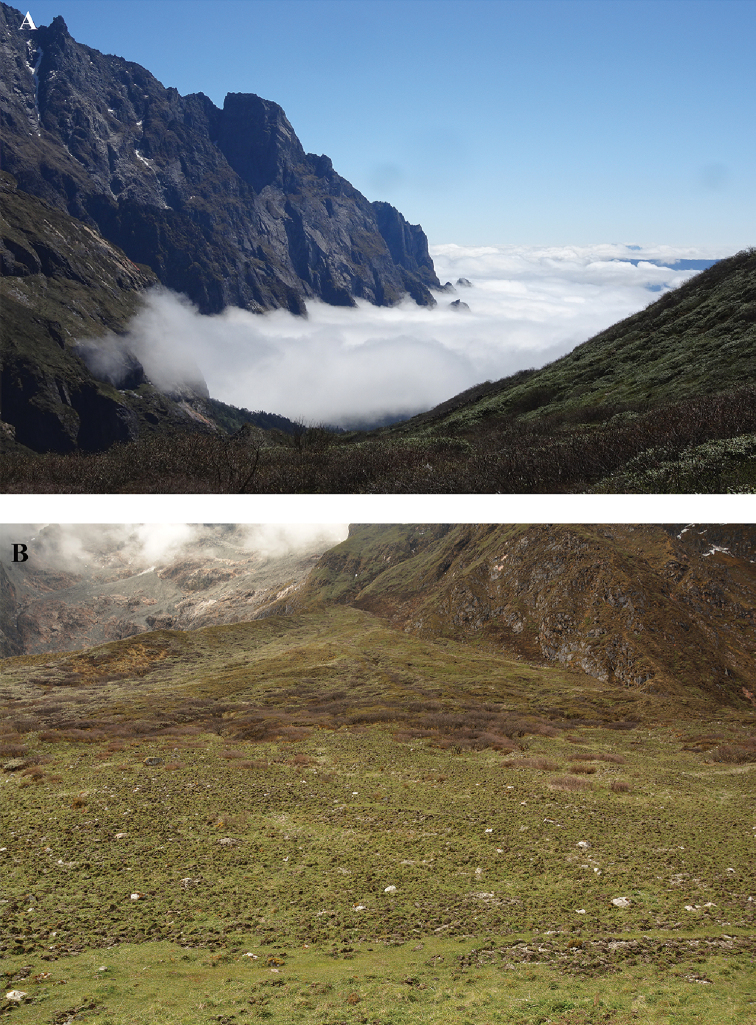
**A** Natural habitat of *Thitarodes shambalaensis* sp. nov. at Yanzigou glacier entrance point, Mt. Gongga, Sichuan, China (photograph credit Meng Li, May 2018) **B** disturbed habitat of *Thitarodes shambalaensis* sp. nov. due to excavation of *T. shambalaensis* pupae, at Haizidang, Yanzigou glacier (photograph credit Wenbin Ju, May 2019).

**Figure 3.**
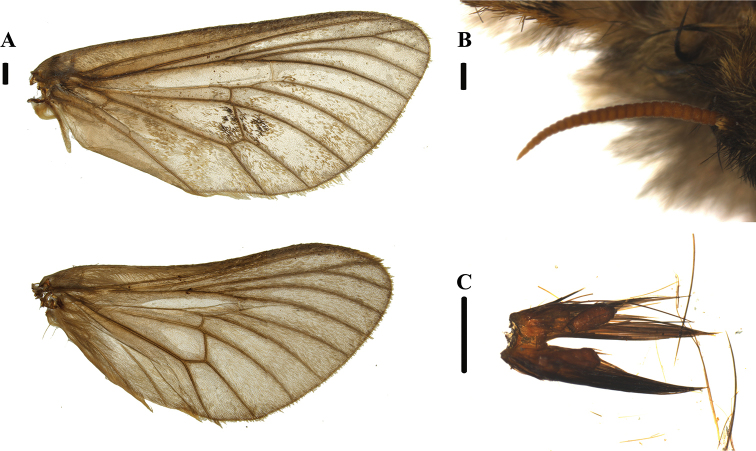
*Thitarodes shambalaensis* sp. nov. **A** forewing (up) and hindwing (bottom) venation from dorsal view **B** antenna, front view **C** labial palp, caudal view, covered under setae. Scale bar: 1 mm.

**Figure 4.**
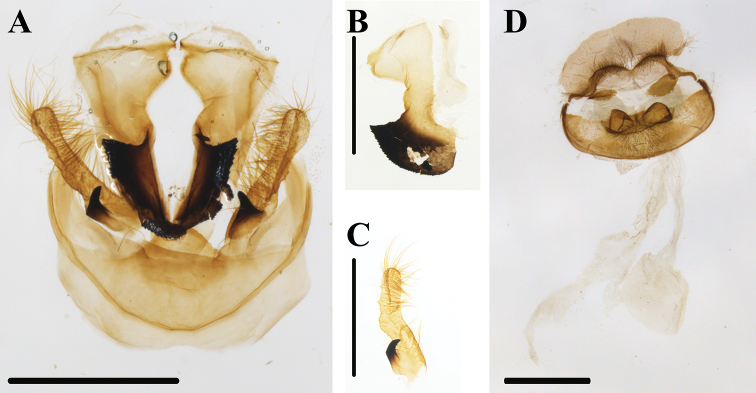
*Thitarodes shambalaensis* sp. nov. **A** male genitalia from holotype, ventral view **B** valva, caudal view **C** pseudotegumen, lateral view **D** female genitalia from paratype. Scale bar: 1 mm.

**Figure 5.**
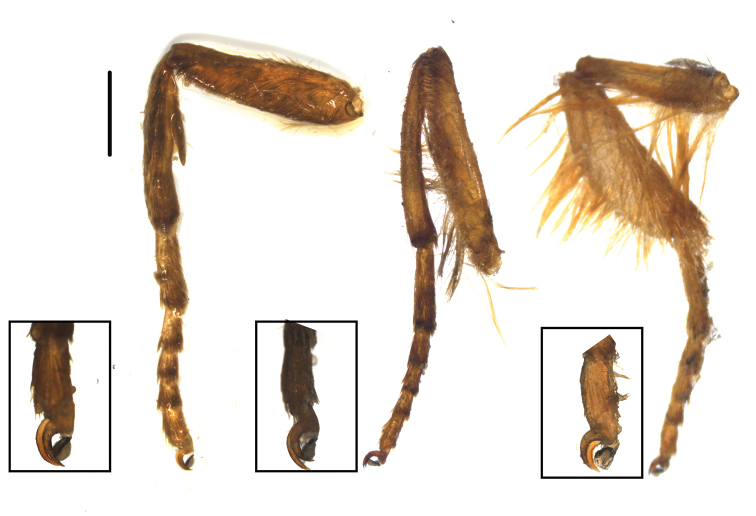
*Thitarodes shambalaensis* sp. nov. Foreleg (left), midleg (middle), hindleg (right) with each aerolium enlarged in black rectangles. Scale bar: 1 mm.

Figs 1 , 2 , 3 , 4 , 5

#### Type material.

***Holotype***: CHINA • ♂; Mt. Gongga, Luding county, at the head of Yanzigou valley (燕子沟), Yangliuping habitat (杨柳坪); 29°41'2.54"N, 101°53'32.24"E; alt. 3892 m; 25–30 Jun. 2016; H. Zuo leg.; MK226958; Sichuan Plant Quarantine Station.

***Paratypes***: CHINA • 2 ♂; same collection data as for holotype • 2 ♂; Mt. Gongga, Luding county, at the head of Yanzigou valley (燕子沟), Haizidang habitat (海子凼); 29°40'17.18"N, 101°53'48.25"E; alt. 3977 m; 25–30 Jun. 2016; H. Zuo leg. • 2 ♀; same collection data as for holotype.

#### Etymology.

From the Sanskrit word शम्भल (Shambala). In Hindu and Tibetan Buddhist tradition, the term refers to a mythical kingdom hidden in the snow mountains. The name refers to the magnificence of the species’ alpine habitat under Mt. Gongga.

#### Description

(based on Holotype). ***General*.** In resting position, forewings fold perpendicular above the sagittal plane of the body and abdomen, completely covering the hindwings. The apex and termen of the two forewings contact each other, while the costa of each forewing extends from the tegula, forming an isosceles. Setae above the thorax form a triangular patch. Wingspan: 44.0 mm (mean = 41.2 mm, SD = 3.5, *N* = 6). Forewing length: 19.6 mm (mean = 19.2 mm, SD = 1.6, *N* = 6), width: 9.9 mm (mean = 9.9 mm, SD = 0.6, *N* = 6). Hindwing length: 16.9 mm (mean = 16.5 mm, SD = 0.8, *N* = 6), width: 12.25 mm (mean = 11.7 mm, SD = 0.9, *N* = 6). Body length: 15.3 mm (mean = 14.7 mm, SD = 1.1, *N* = 6). Thorax width: 3.7 mm (mean = 3.6 mm, SD = 0.2, *N* = 6).

***Head*.** Antenna (Fig. 3B) with 24 annular segments, rust brown, filiform, apical segment acute, surrounded by ocherous setae at base. Black compound eyes. Labial palps (Fig. 3C) short and hidden under dense setae, two-segmented and fused at the base. Other mouthparts absent.

***Body*.** Red-brown. Dense ocherous and yellow setae on thorax. Red and black setae on lateral, ventral and caudal side of the abdomen.

***Legs*.** Fig. 5. All legs setose. Aerolium present on all legs. Foreleg with tibial epiphysis. Hindleg tibia broad and covered with scent-scales.

***Wings*.** Fig. 3A. Jugum present. Forewing grey, with scattered dark brown spots and pale ocherous border. Alternating dark spots on forewing terminal margin. Costa straight. Sc unbranched. R almost parallel to Rs1 + 2. Apex of curvature occurs between Rs1 and Rs2. Rs1 and Rs2 stalked. Rs3 and Rs4 stalked. First half of Rs3 runs parallel to Rs1 + Rs2. R-M weak but visible, concave towards outer margin, reaching Rs4 distal to the bifurcation of Rs3 and Rs4. On forewing two crossveins between CuP and A with basal crossvein almost opposite CuP-CuA2 crossvein, distal crossvein near terminus of CuP. Single crossvein between CuP and CuA2. Vein A reaches dorsum margin. Hindwing grey to transparent; ocherous color on fringe. Venation similar to forewing except both A and CuP reach dorsum margin.

***Male genitalia*.** Fig. 4A–C. From ventral view, a pair of pseudotegumina form an equilateral triangle, with the apex of the triangle pointing ventrally. Dorsal margins of the pseudotegumina flat. Pseudotegumenal arm is strongly sclerotized, distally forming a fan-shaped sclerotized apex, with minute teeth on the lateral margin. Valvae setose, distally lobate, with a shallow longitudinal groove. Base of valve with prominent hook, heavily sclerotized, projecting disto-medially. Saccus forms a median lobe projecting ventrally.

***Female genitalia*** (based on paratype). Fig. 4D. Dorsal plate (tergum IX) dorsally convex to either side of median, setose. Lateral margins dorsally concave with inner edge, with a triangular projection near base of anal sclerites. Anal sclerites subrectangular with rounded margins. Antevaginalis broad with central lobe subdivided into two dorsally rounded knobs, central lobe setose.

#### Diagnosis.

This species has no distinct external sexual dimorphism. Male pseudotegumen triangular with heavily sclerotized pseudotegumenal arms. Pseudotegumenal arms fan-shaped. Valva densely haired with sclerotized, hook-like apex. Venation of *T. shambalaensis* is similar to that of *T. namnai* Maczey, 2010 in Nepal, but in *T. shambalaensis* both A and CuP reach dorsum margin on the hindwing. *Thitarodes markamensis* (Liang, Li & Shen, 1992) has also reached the degree of heavy sclerotization on the pseudotegumenal arms, but the two species can be distinguished by differences in venation (where MR furcation in *T. shambalaensis* is distal to the furcation Rs3 and Rs4) and an inflation on the posterior margin of the saccus. Sclerotization at the ventral base of the valvae with a spinal projection is also present in other species of *Thitarodes*, such as in *T. jiachaensis* Zou, 2011 and *T. sejilaensis* Zou, 2011, but the spinal projection is less curved and the setose lobe of the dorsal side of the valvae is more elongated in *T. shambalaensis*.

**Figure 6.**
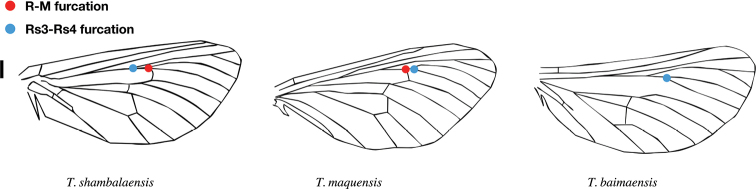
Diagnostic character of *Thitarodes shambalaensis* sp. nov. in forewing venation in comparison to other species of *Thitarodes*. Scale bar: 1 mm.

#### Remarks.

**Figure 7.**
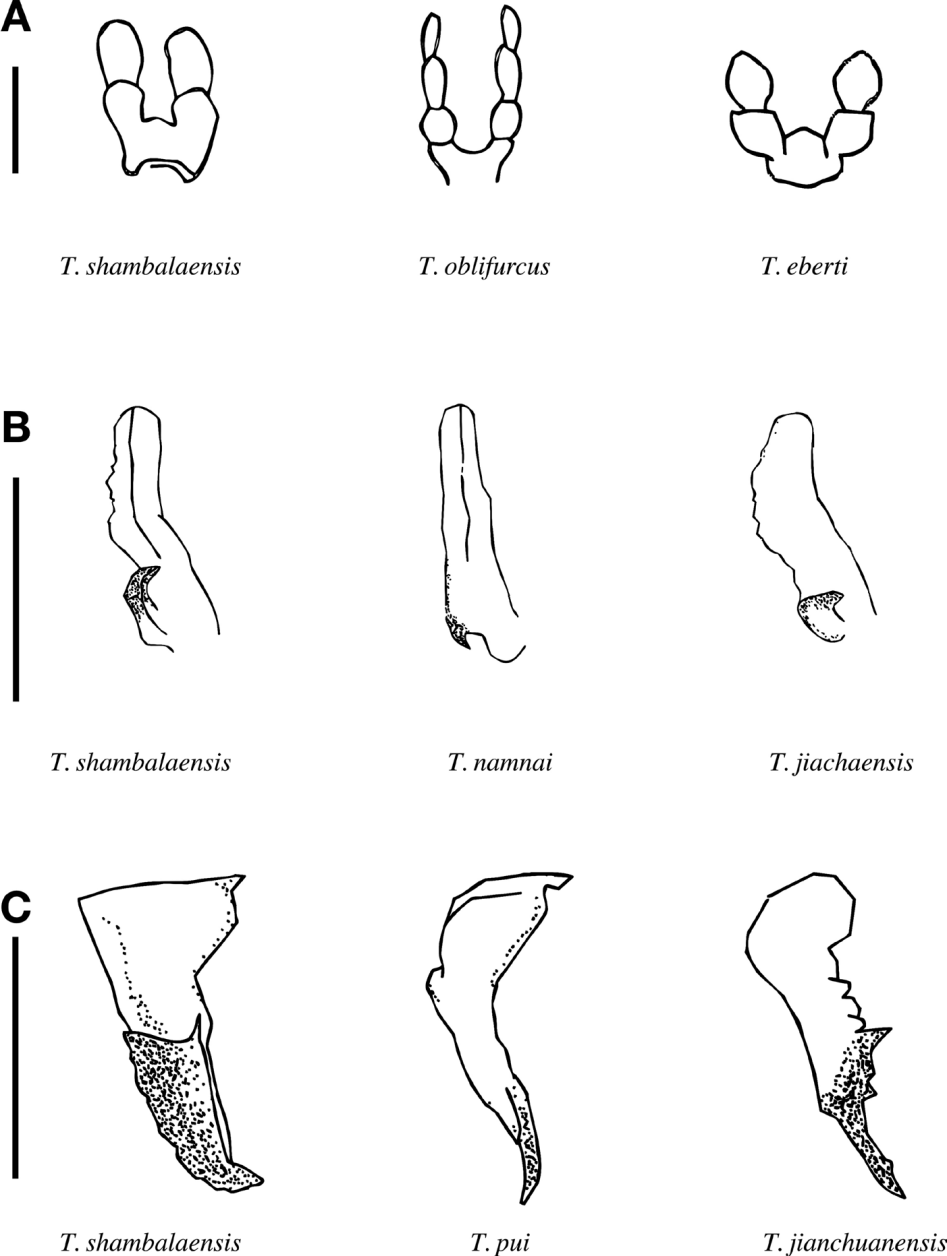
Diagnostic characters of *Thitarodes shambalaensis* sp. nov. in comparison to other species of *Thitarodes***A** labial palp **B** valva **C** pseudotegumen. Scale bar: 1 mm.

With the exception of those by Ueda (2000), descriptions of *Thitarodes* species rarely include an illustration of the labial palp. Mouthparts are often difficult to observe and sometimes reduced (or absent) in *Thitarodes*, yet when illustrated, have always shown notable differences between species (Fig. 7A). We encourage in future description of species in *Thitarodes* to include an illustration of the labial palps, as it might serve as a trait useful in resolving clade relationships within the genus.

#### Phenology.

Late instar larvae, sometimes already infected by *O. sinensis*, can be found as early as mid-May, about 30 cm under soil. Pupae can be found starting early June in soil. Adults appear in a week in late June.

#### Ecology.

The species is found in several high elevation (3400–3800 m) alpine grassland along the glaciers of eastern Mt. Gongga (Fig. 2). Larvae are ground-boring generalist plant root eaters. Common flowering plants in in the grassland include *Saxifraga*, *Rhodiola*, *Polygonum*, *Corydalis*, *Primula*, *Potentilla* and *Anemone*; *Salix* and *Rhododendron* bushes are also present. Adults are not attracted by light and no mating flight has been observed.

#### Conservation threat.

The species is the host of *O. sinensis*. Caterpillar fungus collection has for decades provided income for local people in eastern Mt. Gongga. The traditional method of collection has not had a discernible impact on populations of *T. shambalaensis*, but since 2016, medical pharmaceutical companies have begun buying *T. shambalaensis* pupae from local people for a commercial caterpillar fungus farming project. Many *T. shambalaensis* pupae have been excavated from their habitat each year, sold and transferred to commercial breeding stations. Local people have expressed concern at such habitat exploitation. When we visited the Haizidang habitat in 2019, this alpine grassland had been completely uprooted due to pupae excavation (Fig. 2B).

## Results

### Validation with CO1 and RAD‐Seq

**Figure 8.**
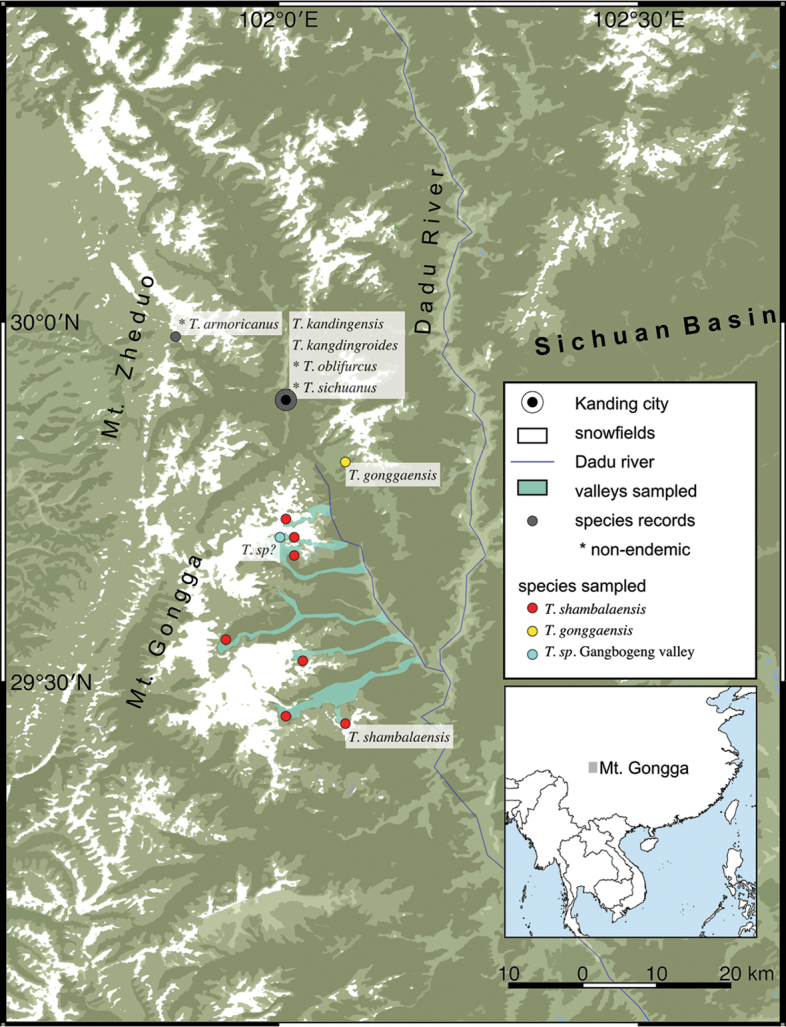
The distribution of *Thitarodes* species sampled in this study and historical records of *Thitarodes* species in the Kangding – Mt. Gongga region.

**Figure 9.**
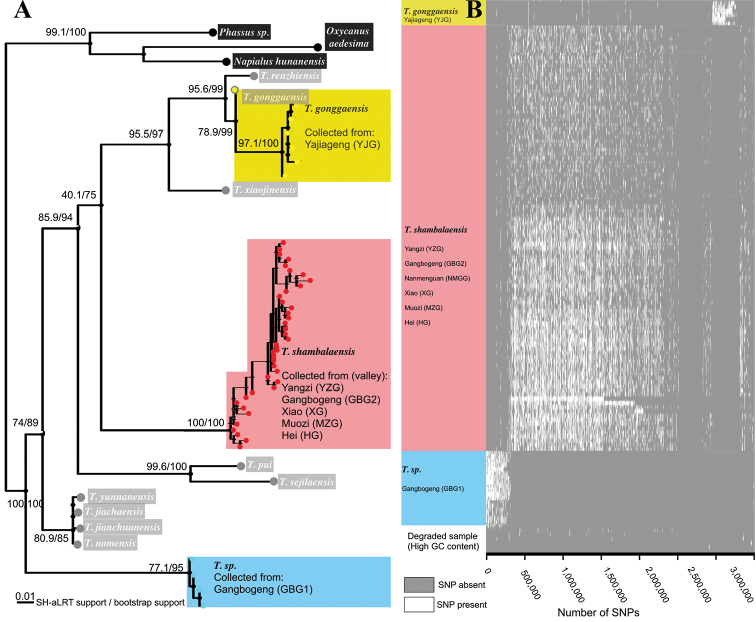
**A** Maximum Likelihood tree of CO1 sequences of species sampled in this study (yellow, red and blue dots) and other known species of the genus *Thitarodes* (grey dots). Outgroups are in black dots. The corresponding position of the genus *Ahamus* designated by Zou et al. (2010) is labeled **B** RAD-Seq SNP coverage of samples from three species (and degraded samples).

COI sequences of 48 *Thitarodes* samples collected from six glacial valleys were successfully sequenced (Figs 8, 9A, Suppl. material 1: Table S1). The best model for the parsimony tree assessed by the BIC value determined by ModelFinder (Kalyaanamoorthy et al. 2017) is “K3Pu+F+I+G4”. All *Thitarodes* sequences form a highly supported monophyletic group, sister to non-*Thitarodes* outgroups. These samples cluster into three highly supported clades in the phylogeny when other available CO1 sequences of *Thitarodes* is taken into account (Fig. 9A): (1) All samples from the Yajiageng valley (GenBank accession number MK226959) form a monophyletic clade that exhibits less than 3% divergence from a deposited sequence of *T. gonggaensis* (Fu & Huang, 1991) in GenBank by Shi et al. (2016). (2) All samples collected at a lower altitude habitat at Gangbogeng valley (GenBank accession number MK226960) formed a highly supported clade with no known sister group that is less than 10% divergent. (3) The rest of the samples, including all samples collected at the habitat of *T. shambalaensis* and other samples collected in six different glacial valleys, form a highly supported clade (GenBank accession number MK226958) with more than 5% divergence from any known sequence of *Thitarodes* species.

A total of 128 out of 134 samples from seven glacial valleys was successfully sequenced according to the RAD-seq protocol (Davey and Blaxter 2010); this generated on average 4,945,132 reads (SD = 2,841,511), and 582,986 SNPs (SD = 357,473) per sample. A total of 3.9 million SNPs was counted (Fig. 9B, Suppl. material 2: Table S2). Hierarchical clustering of reads coverage showed three groups of samples sharing SNPs: (1) All six samples collected from Yajiageng valley share 0.3 of the 3.9 million SNPs. These samples’ COI sequences are similar to that of *T. gonggaensis* (GenBank accession number MK226959). (2) All 16 samples from the lower altitude habitat at Gangbogeng valley, collected in both 2016 and 2017, share 0.3 of the 4 million SNPs not present in any other samples. The COI sequences of these samples (GenBank accession number MK226960), form a unique group with no phylogenetically close sister taxon. (3) All 106 samples collected in valleys across the eastern side of Mt. Gongga share the majority of the 4 million SNPS that are not shared by (1) and (2). The COI sequence of this group corresponds to that of *T. shambalaensis* described in this study (GenBank accession number MK226958).

### Summary tree

A total of 538 sequences (excluding three sequences from non-*Thitarodes* outgroups) was used to construct a summary phylogenetic tree (Suppl. material 3: Table S3, 142 CO1 sequences,163 Cytb sequences, 33 CO2 sequences, 103 wingless (wg) sequences, 6 mitogenomes, 91 multiple locus haplotypes (MLH) based on CO1). These sequences represent genetic material from at least 175 individuals belonging to the genus *Thitarodes* (excluding MLH sequences). Of the 175 individual samples only 42 have a species name associated, and only eight of which, including two from this study, can be verified in a publication. The best model for parsimony tree by BIC value determined by ModelFinder (Kalyaanamoorthy et al. 2017) is “TIM2+F+I+G4”. All sequences from known *Thitarodes* moth individuals and caterpillar fungus sclerotium extractions form one monophyletic group, clearly separated from the other Hepialidae genera *Napialus* Chu & Wang, 1985, *Oxycanus* Walker, 1856 and *Phassus* Walker, 1856. MLH sequences from Quan et al. (2014a) potentially overlap with individual CO1 sequences provided in Quan et al. (2014b). It is unclear whether Quan et al. (2014b) provided individual-level or population-level consensus sequences, but their list of localities is partly identical to Quan et al. (2014a). These MLH sequences (91) were dropped from the analysis. Of the resulting 184 individual samples, 154 have their closest sister group within 0.025 genetic distance from another individual. Cytb sequences of two samples, both labeled *T. armoricanus*, are placed into two distinct clades. The structure of the summary tree is similar to the phylogenetic tree generated from only CO1 sequences, except with the placement of samples collected from Gangbogeng valley.

**Figure 10.**
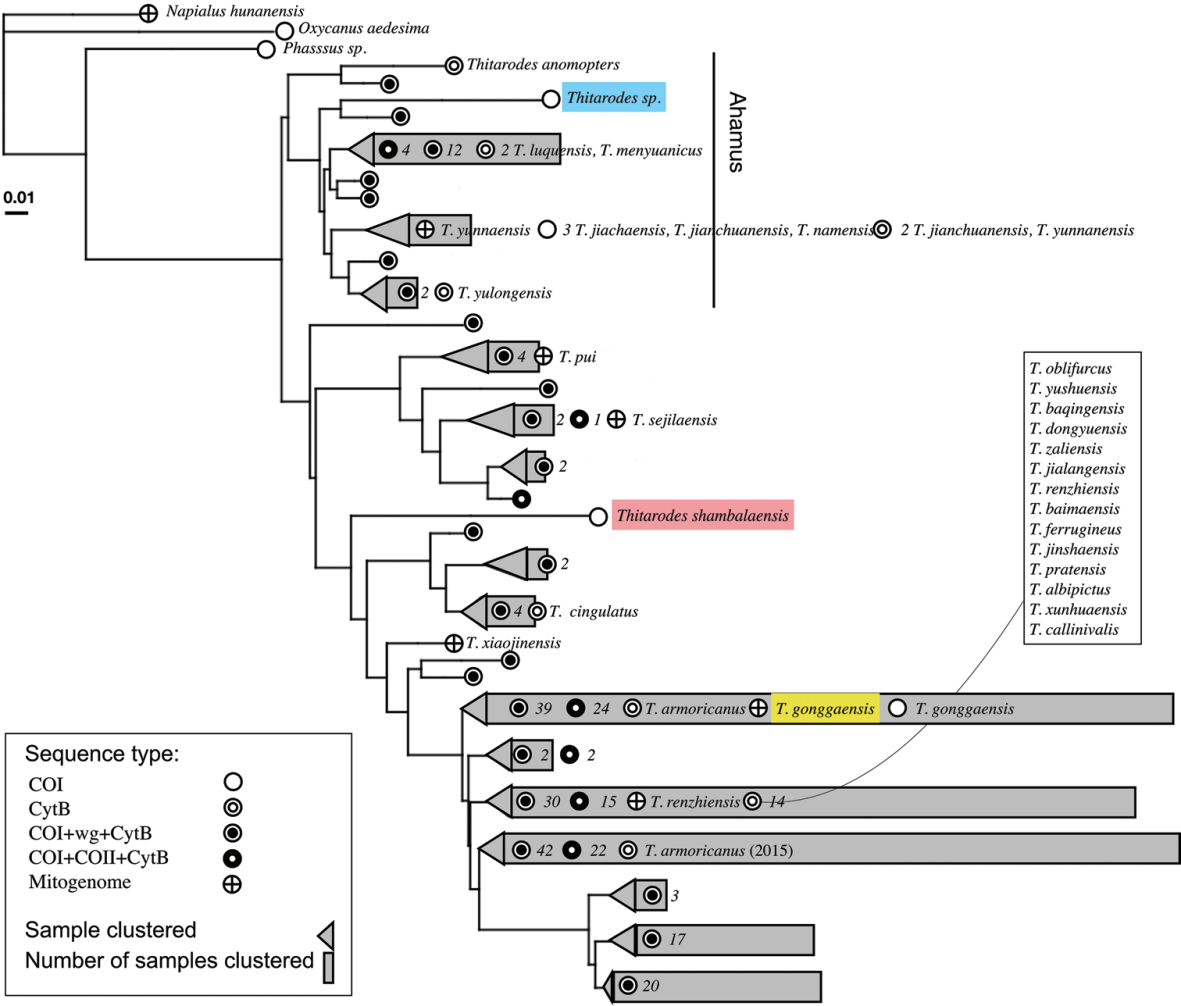
Summary tree for all known species of *Thitarodes*. Samples in this study are labeled in color bars (blue, red and yellow). Each tip represents a cluster of all samples that are within 2.5% genetic distance from each other, the length of the grey bar represents the number of individual samples falling into the cluster. Numbers after the dots enumerate the number of samples that fall into a particular sequence type. All named individual samples are labeled. The genus *Ahamus* as designated by Zou et al. (2010) is labeled.

## Discussion

### Phylogenetic position of *T. shambalaensis*

Our analysis shows that *T. shambalaensis* is not only morphologically, but also phylogenetically distinct from other known species in the genus *Thitarodes*. Even according to the generic description by Zou et al. (2010), it is still nested amongst other known *Thitarodes* species. Maczey et al. (2010) suggested that based on morphology, it is possible that many currently described species (such as *T. gonggaensis* (Fu & Huang, 1991), *T. jialangensis* (Yang, 1994), *T. namensis* (Chu & Wang, 2004), *T. namlinensis* (Chu & Wang, 2004), and *H. hainanensis* Chu & Wang, 2004) are subspecific variations of a single widespread species ranging from Sichuan to Nepal, and this hypothesized species would include the later described *T. jiachaensis* and *T. shambalaensis*. Our phylogenic analysis shows that these latter two taxa are distinct. Although the genitalic structure and wing venation of *T. shambalaensis* is most similar to *T. jiachaensis*, the two taxa are phylogenetically distant.

Some other *Thitarodes* species also possess triangular pseudotegumina with fan-shaped, indented sclerotization at the pseudotegumenal arms, such as *T. jialangensis* (Yang, 1994), *T. pratensis* (Yang, 1994), *T. callinivalis* (Liang, 1995), *T. litangensis* (Liang, 1995), *T. kangdingensis* (Chu & Wang, 1985), and *T. markamensis* (Liang, Li & Shen, 1992). Although morphologically distinct from *T. shambalaensis* (e.g., *T. jialangensis* Yang, 1994 has a dark forewing without spots; *T. pratensis* has an elongated 23^rd^ antenna segment and orange eyes), it is possible that these species, along with *T. shambalaensis*, are subspecific variations of a single widespread species ranging across the Hengduan Mountains. This hypothesis could not be further tested without a thorough revision of the genus.

Our phylogenies are largely consistent with the subdivision of the genus into *Thitarodes* and *Ahamus* by Zou et al. (2010): the latter genus, containing species such as *T. jianchuanensis* and *T. yunnanensis* (Yang, Li & Shen, 1992), form a monophyletic group in both our CO1 phylogeny and our summary phylogeny. One major inconsistency between our results and Zou et al. ’s (2010) treatment is that both *T. jiachaensis* and *T. namensis* in our phylogeny would fall into the clade of *Ahamus*: this is inconsistent because the defining morphological trait for genus *Ahamus* (Latin for “no hook”) is the lack of a hook-like structure at the ventral side of the valvae, which *T. jiachaensis* and *T. namensis* clearly possess. We note that since “no hook” is a plesiomorphic trait, it is problematic to use it to justify a monophyletic group within *Thitarodes* group (i.e., *Ahamus*). It is also possible, since CO1 sequences of *T. jiachaensis* and *T. namensis* used in this study are unpublished, that the assignment of the samples to these sequences are in error. We encourage other workers in the field to provide evidence regarding whether *T. jiachaensis* and *T. namensis* phylogenetically fall within the genus *Ahamus* (Zou et al. 2010) and whether there are additional, closely related groups to *T. shambalaensis* described in this study.

### An undescribed taxon to test the classification of Zou et al. (2010)

Phylogenetic placement and SNP coverage visualization from our study both suggest that some larvae and pupae samples collected from a lower elevation habitat in Gangbogeng valley across two consecutive years belong to an undescribed taxon (*T. shambalaensis* has been collected along this same valley, but in a habitat at a higher altitude). Our attempts to collect adults of this unknown taxon from 2016 to 2018 have not been successful, nor is it found in any other valley that we sampled. The limited range of this taxon compared to the parapatric and relatively widespread *T. shambalaensis* and its evolutionary history is intriguing. If the group is indeed a member of the genus *Ahamus*, as defined by Zou et al. (2010), we predict that the adults should not possess a hook-like structure on the ventral sides of its valvae.

### *Thitarodes* around Kangding

CO1 sequences of our samples collected at Yajiageng valley are closely related (within 2% divergence) to known sequences labeled as *T. gonggaensis* in the study of Shi et al. (2016). The type locality of the species is “Kangding” (Fu and Huang 1991); Shi et al. (2016) collected the sample at Gonggashanxiang (29°32'24.0"N, 101°35'24.0"E), 65 km south west of Kangding. The locality of Shi et al. (2016) is also 57 km southwest of the samples of *T. gonggaensis* collected in this study, thus explaining the divergence with samples collected in our studies.

According to Wang and Yuo (2011), four other species have “Kangding” as the type locality or have been recorded in “Kangding”: *T. kangdingroides* (Chu & Wang, 1985), *T. kangdingensis* (Chu & Wang, 1985), *T. oblifurcus* (Chu & Wang, 1985), and *T. sichuanus* (Chu & Wang, 1985). It is unlikely that these samples were collected at Kangding city (30°01'21.1"N, 101°57'27.6"E). Although the city is one of the most prominent trading centers for caterpillar fungus, it is located at a relatively low elevation (2,500 m) and is not a known habitat of caterpillar fungus. These species, like samples of *T. gonggaensis* in this study and in Shi et al. (2016), were most likely collected in the mountains around Kangding city. Specimens of these four other species are difficult to access and no genomic data has been published at this time.

The type species of the genus, *T. armoricanus*, is also recorded as collected (and reared) by Tao et al. (2015), at Mt. Zheduo, 15 km northwest of Kangding, although no specimens have been dissected to verify the identification. This suggests the existence of at least eight species of *Thitarodes* (counting *T. shambalaensis* and the unknown species from Gangbogeng valley sequenced in this study) in the vicinity of Kangding-Mt. Gongga region, six of which are endemic. To our knowledge, much of the caterpillar fungus habitat around the Kangding-Mt. Gongga area, especially toward the western and north-western slope of Mt. Gongga, has not been thoroughly surveyed. We would expect more species to be discovered in this region following a systematic survey. It is hypothesized that vicariance generated by the mountain topology of the Hengduan mountains, of which Kangding is part, contributed to the rate of speciation of this biodiversity hotspot (Xing and Ree 2017). Regarding the genus *Thitarodes*, species diversity and endemism is not unique to the Kangding-Mt. Gongga region: the snow mountains of Yulong, Meili, Laojun in Yunnan, also part of the Hengduan mountains, is habitat to highly endemic species described to be living in close proximity (Yang 1994; Liang 1995).

### Species delineation with CO1 and SNP coverage

A common concern in any study of the population genetics of non-model organisms is whether the analyzed samples come from different populations of the same species, or whether multiple species are involved. In our study, we still need to verify whether all *Thitarodes* samples collected across the eastern slope of Mt. Gongga are the same species. Without molecular evidence, this is difficult to do, since adult *Thitarodes* samples are difficult to identify, and most samples are obtained in larval or pupal forms, which are not sufficiently taxonomically informative for species delineation.

CO1 sequences show that our samples cluster into three clades, with inter-clade divergence falling below the 3.78% commonly observed in populations within a species, and intra-group divergence falling within the 11.06 % range of species within the same genus boundary (see review of CO1 sequence divergence by Kartavtsev 2011). We consider this strong evidence supporting for the existence of three distinct species, however, this level of interspecific (intra-population) divergence is not sufficient to distinguish patterns of population evolution history (e.g., evolutionary relationships between population of *T. shambalaensis* in Yanzigou valley and *T. shambalaensis* in Gangbogeng valley in association with the history of glaciation).

RAD‐Seq has traditionally been considered useful in population level studies, while species-level divergence would reduce the amount of shared SNP coverage across species (Davey and Blaxter 2010; Cariou et al. 2013; Eaton et al. 2017). Our results suggest that RAD‐Seq provides enough SNPs within species to further analyze the population history of each species but is not informative in analysis at the interspecific level, as samples with SNP data form three distinct clusters, but these are not shared among all samples. The clustering of those taxa with SNP data supports the CO1 species delineation indicating three main lineages.

We conclude that in this study, both the phylogeny based on CO1 sequences and the visualization of genome-wide SNP coverage provide evidence for the presence of three species in the eastern slopes of Mt. Gongga: *T. shambalaensis* occupies most valleys in the eastern slopes of Mt. Gongga; *T. gonggaensis* is found in Yajiageng valley; another undescribed species is isolated at a low elevation habitat at Gangbogeng valley.

### *Thitarodes* phylogeny

Our summary tree shows that the genus *Thitarodes* is monophyletic. All known moth sequences extracted from caterpillar fungus sclerotia, despite the difficulty of assigning them to discrete species, nevertheless cluster within the genus *Thitarodes*.

Among the 538 sequences from 184 individuals of the genus *Thitarodes*, only 42 could be associated with a species name, and only eight of these are verified in publications. Of the 54 species summarized by Chu et al. (2004), only 39 have matching genetic information, most of which are unpublished Cytb sequences. 140 of the individuals are caterpillar fungus sclerotia samples, 66 of which are not within 2.5% phylogenetic distance of a known species. This suggests that many additional taxa remain to be identified and described.

In cases where multiple sources of sequences are all labeled as the same species, species identification has shown to be quite consistent: the Cytb sequence of *T. yunnanensis* matches to the mitogenome of *T. yunnanensis*, the Cytb sequence of *T. jianchuanensis* clusters together with CO1 sequence of *T. jianchuanensis* (as both are aligned with other mitogenomes), the CO1 sequence of *T. gonggaensis* matches its mitogenome, and the CO1 sequence of *T. renzhiensis* matches its mitogenome. Slight variations exist between the segment sequences and their corresponding flanking regions on the mitogenome, but we could find no phylogenetic inconsistencies. The only exception is that the two entries of the Cytb sequence of the type species of the genus, *T. armoricanus*, are distinctly different. While we have reason to believe that the type specimen of this sample was collected around Kangding (Chu 1965), Wang and Yao (2011) noted that this species ranges from Gansu, Tibet to Xinjiang. As we discussed in the previous section on the endemism of *Thitarodes* in the Hengduan mountains, we are of the opinion that the range estimate *T. armoricanus* of Wang and Yao (2011) includes undescribed cryptic species that have distinctive Cytb sequences, as revealed in our phylogenetic analysis.

Our summary tree also reveals inadequate sampling in many clades. When clustered by a 2.5% genetic distance (what we consider to be a reasonable threshold for inter-species variation, see Kartavtsev 2011), only 30 genetic clusters were recovered. Out of the 184 individual samples, 154 fall into 15 out of the 30 clusters; the other 15 clusters are known by either a single “orphan taxon” (*T. shambalaensis*, *T. anomopterus*, *T. xiaojinensis* (Tu, Ma & Zhang, 2009), *T.* sp. from Gangbogen in this study) or just a few sequences from a caterpillar fungus sclerotium sample. Additional research will help to elucidate the nature of these lone taxa. It is likely that “close sisters” of these taxa might be found if all known species of *Thitarodes* could be sequenced.

The bulk of the sampling effort so far has focused on only half of the known diversity of *Thitarodes*. This uneven phylogenetic sampling is not simply the result of relying on sequences of caterpillar fungus sclerotia, which have no morphological species description. The uneven sampling problem extends to the phylogeny of named species as well: the reference sequences of 39 out of the 42 named species fall into one of those 30 2.5%-distance clusters. One such cluster (including *T. renzhiensis*, *T. oblifurcus*, etc.) includes 17 known species! Most of these 17 species are known by one Cytb sequence only, which might be the result of sampling a particularly invariant region of the mitogenome. However, this issue has not been brought to attention in previous attempts to summarize genetic data for the genus (Zou et al. 2010, 2017). We noticed that in the comprehensive sampling across known range of caterpillar fungus, Zhang et al. (2014) only recovered seven well-supported clades and two “orphan taxa”. It is unclear by what genetic distance Zhang et al. (2014) have considered to be a clade, but their estimate might be a closer estimate of the number of phylogenetically well-established species in *Thitarodes*.

To summarize, our current understanding of the genus *Thitarodes* derives from three major sources:

1 The analysis of samples of caterpillar fungus sclerotia, and sometimes just *Thitarodes* larvae or pupae, has provided crucial information about the habitat and ecology of the species in the genus, but often fails to provide information about the morphology of the *Thitarodes* adult. We encourage workers in this field to include more detailed descriptions of the habitats where samples of caterpillar fungus are collected (including vegetation, climate and soil type), and make an effort to understand adult *Thitarodes* biology in these known habitats.

2 Many described species have types deposited in institutions around China; earlier descriptions of these samples often do not include photos of wing venation and genitalia. Revisions of these taxa with updated photos and molecular data for comparative analysis are critical.

3 New descriptions of species in the group (see Jiang et al. 2016), and NGS-based analysis of several species of the group (see Guo et al. 2016; Li et al. 2016), should provide reference sequences along with genitalia dissections, so that any known species can be linked to its position in the molecular phylogeny.

## Supplementary Material

XML Treatment for Thitarodes shambalaensis

## Supplementary materials

Supplementary material 1 Table S1. Sequences used in Fig. 9A Data type: molecular data https://binary.pensoft.net/file/352701This dataset is made available under the Open Database License (http://opendatacommons.org/licenses/odbl/1.0/). The Open Database License (ODbL) is a license agreement intended to allow users to freely share, modify, and use this Dataset while maintaining this same freedom for others, provided that the original source and author(s) are credited.Zhengyang Wang, Hailing Zhuang, Min Wang, Naomi E. Pierce

Supplementary material 2 Table S2. Samples sequenced shown in Fig. 9B Data type: molecular data https://binary.pensoft.net/file/352702This dataset is made available under the Open Database License (http://opendatacommons.org/licenses/odbl/1.0/). The Open Database License (ODbL) is a license agreement intended to allow users to freely share, modify, and use this Dataset while maintaining this same freedom for others, provided that the original source and author(s) are credited.Zhengyang Wang, Hailing Zhuang, Min Wang, Naomi E. Pierce

Supplementary material 3 Table S3. Sequences used in Fig. 10 Data type: molecular data https://binary.pensoft.net/file/352703This dataset is made available under the Open Database License (http://opendatacommons.org/licenses/odbl/1.0/). The Open Database License (ODbL) is a license agreement intended to allow users to freely share, modify, and use this Dataset while maintaining this same freedom for others, provided that the original source and author(s) are credited.Zhengyang Wang, Hailing Zhuang, Min Wang, Naomi E. Pierce
